# Enhancing the quality and reproducibility of research: Preferred Evaluation of Cognitive and Neuropsychological Studies - The PECANS statement for human studies

**DOI:** 10.3758/s13428-025-02705-3

**Published:** 2025-05-30

**Authors:** C. Costa, R. Pezzetta, E. Toffalini, M. Grassi, G Cona, C Miniussi, P. J. Bauer, S. Borgomaneri, M. Brysbaert, C. D. Chambers, N. Edelstyn, A. Eerland, S. J. Gilbert, M. A. Nitsche, R. A. Poldrack, A. Puce, K. R. Ridderinkhof, T. Y. Swaab, C. Umiltà, M. Wiener, C. Scarpazza

**Affiliations:** 1 Department of General Psychology, University of Padua, Padua, Italy; 2 IRCCS San Camillo Hospital, Via Alberoni 70, Venice, Italy; 3 Center for Mind/Brain Sciences CIMeC, University of Trento, Rovereto, Italy; 4 Department of Psychology, Emory University, Atlanta, GA USA; 5 Centro studi e ricerche in Neuroscienze Cognitive, Dipartimento di Psicologia “Renzo Canestrari”, Alma Mater Studiorum Università di Bologna, Cesena Campus, Cesena, Italy; 6 Department of Experimental Psychology, Ghent University, Ghent, Belgium; 7 Cardiff University Brain Research Imaging Centre, Cardiff, CF24 4HQ UK; 8 School of Sciences, Bath SPA University, Bath, UK; 9 Behavioural Science Institute, Radboud University, Nijmegen, The Netherlands; 10 Institute of Cognitive Neuroscience, University College London, London, UK; 11 Department Psychology and Neurosciences, Leibniz Research Centre for Working Environment and Human Factors, Dortmund, Germany; 12 Bielefeld University, University Hospital OWL, Protestant Hospital of Bethel Foundation, University Clinic of Psychiatry and Psychotherapy and University Clinic, Bielefeld, Germany; 13 German Center for Mental Health (DZPG), Bochum, Germany; 14 Department of Psychology, Stanford University, Stanford, CA USA; 15 Department of Psychological and Brain Sciences, Indiana University, Bloomington, IN USA; 16 Department of Psychology, University of Amsterdam, Amsterdam, the Netherlands; 17 Department of Psychology, University of California, Davis, CA USA; 18 Department of Psychology, George Mason University, Fairfax, VA USA

**Keywords:** Open science, Reproducibility, Replicability, Guidelines, Transparency

## Abstract

Are scientific papers providing all essential details necessary to ensure the replicability of study protocols? Are authors effectively conveying study design, data analysis, and the process of drawing inferences from their results? These represent only a fraction of the pressing questions that cognitive psychology and neuropsychology face in addressing the “crisis of confidence.” This crisis has highlighted numerous shortcomings in the journey from research to publication. To address these shortcomings, we introduce PECANS (Preferred Evaluation of Cognitive And Neuropsychological Studies), a comprehensive checklist tool designed to guide the planning, execution, evaluation, and reporting of experimental research. PECANS emerged from a rigorous consensus-building process through the Delphi method. We convened a panel of international experts specialized in cognitive psychology and neuropsychology research practices. Through two rounds of iterative voting and a proof-of-concept phase, PECANS evolved into its final form. The PECANS checklist is intended to serve various stakeholders in the fields of cognitive sciences and neuropsychology, including: (i) researchers seeking to ensure and enhance reproducibility and rigor in their research; (ii) journal editors and reviewers assessing the quality of reports; (iii) ethics committees and funding agencies; (iv) students approaching methodology and scientific writing. PECANS is a versatile tool intended not only to improve the quality and transparency of individual research projects but also to foster a broader culture of rigorous scientific inquiry across the academic and research community.

## Introduction

The prevalent structure of scientific papers follows the so-called IMRAD format: Introduction, Methods, Results, and Discussion. Originating from the report style of the microbiologist Louis Pasteur (Day, 1989) and later adopted by the entire scientific community (Council of National Library and Information Associations, 1979), IMRAD facilitates the writing, reading, and assessment of the scientific content of a research report. Standards like IMRAD are essential in ensuring that empirical studies can be replicated and reproduced. By providing a structured framework, these standards help authors and readers to locate the relevant information in a research report. This makes it possible to repeat a study using the same methods and data to achieve similar results (reproducibility) or to obtain comparable results when conducting a study using different data but following the same procedures described in the original research (replicability).

Despite the extensive use of standards such as IMRAD, it is well established that contemporary research in psychology and neuroscience suffers a crisis of confidence (Nosek et al., 2022; Pashler & Wagenmakers, 2012). For instance, to provide empirical grounding to the debate on replicability, Nosek et al. (2022) summarized evidence regarding this topic within the field of psychological science. The results indicate varying degrees of success for both systematic and multi-site replications (Camerer et al., 2018; Open Science Collaboration, 2015; Soto, 2019). Among 307 replications considered, 64% reported statistically significant evidence in the same direction, with effect sizes 68% as large as in the original studies. As concluded by the authors, replicability challenges are observed in almost all fields of research that have undergone systematic examination.

The origins of this crisis are diverse and include both endogenous and exogenous factors related to the behavior and environment of scientists (Wicherts et al., 2016; Smaldino & McElreath, 2016). Some of the roots of the crisis can be found in scientific articles themselves, which often lack essential information necessary to judge the scientific soundness of the report, particularly regarding the data (Simmons et al., 2011; see Anvari & Lakens, 2018 for a thorough discussion), as well as the minimum information required for replicating the protocol (Simmons et al., 2011).

Indeed, each section of a research article should contain appropriate information, and it may be convenient for both authors and readers to rapidly identify whether this information is included in the written paper. For example, the Introduction should present the specific problem under investigation and describe the type of research planned. In hypothesis-driven research, it is crucial to state the hypothesis and to identify which outcomes would disconfirm it, allowing for the study itself or future direct replications to potentially falsify the hypotheses (Nosek & Errington, 2020). The Methods section is particularly critical as it should list all relevant information necessary for a direct replication of the experiment (see Zwaan et al., 2018, for a discussion about direct and indirect replication, and Nosek & Errington, 2020, for a pragmatic definition of direct replication). Authors should explicitly describe which key elements of the study drive and modulate the effect under investigation (Grassi et al., 2021). The (lack of) statistical power of studies is often addressed as one of the major problems in contemporary research in cognitive psychology and neuropsychology (Button et al., 2013; Cohen, 1962; Sedlmeier & Gigerenzer, 1992; Szucs & Ioannidis, 2017, 2020). Authors should report whether a power analysis has been performed, provide details for third parties to assess its correctness, or explain any other decision that may lead to a specific sample size (Lakens, 2022). When reporting Results, if inferential statistics are planned, p-values should be reported in their exact values at least up to the third decimal point (therefore, unless it is <.001) (APA, 2024), rather than solely indicating if they fall below a significance threshold, and significance levels should be reported along with all the key elements necessary to understand the statistics, such as the value of the statistic itself and the degrees of freedom (see Greenland et al., 2016 for explanations of *p* values, confidence intervals and power misinterpretations). Contemporary papers should also provide other relevant information, such as whether the study and/or analysis plan was pre-registered and if there were any deviations from the pre-registered study design, conduction, and analysis plan, which is often absent in papers (e.g., Claesen et al., 2021). Additionally, authors should indicate whether data and materials are openly available, and if so, provide information on how these materials can be accessed (Wicherts et al., 2006). Altogether, these key sources of information contribute to the trustworthiness of the study (e.g., Kidwell et al., 2016).

Given the substantial amount of relevant information that authors need to report, and readers should be aware of, we believe that a checklist could assist authors, readers, editors, and reviewers in ensuring that a research report includes all necessary information to guarantee clarity, rigor, accurate evaluation, interpretation and, if relevant, replication. Some instruments have already been developed (e.g., STROBE guidelines for observational studies in epidemiology (Von Elm et al., 2007), CONSORT guidelines for randomized trials (Schulz et al. 2010), CARE guidelines for case reports (Riley et al. 2017), PRISMA guidelines for systematic reviews (Page et al., 2021)). All these guidelines are included in the EQUATOR network (https://www.equator-network.org/), a global organization focused on enhancing the quality of the scientific literature and promoting transparent reporting of health research studies to support and promote reproducibility and usefulness. Yet, the fields of cognitive psychology and neuropsychology lack a specialized checklist tailored to meet the unique demands of these disciplines. A step in this direction is undoubtedly represented by the work published by Aczel et al. (2020), who introduced a consensus-based instrument to improve the transparency of research reports in social and behavioral research, and by the APA Style Journal Article Reporting Standards (APA Style JARS; https://apastyle.apa.org/jars), a set of standards including information on what should be included in a quantitative, qualitative, or mixed-methods research report, designed for authors, reviewers, and editors to enhance rigor in peer-reviewed journal articles. Additionally, a checklist for assessing the transparency of methods in a scientific report was recently published (Zogmaister et al., 2024). However, given the increasing refinement and sophistication of cognitive manipulations in experimental designs, and the increasing significance of cognitive psychology and neuropsychology in healthcare research (Wallin et al. 2018), there is an urgent demand for guidelines tailored to address the unique characteristics of these disciplines. While existing instruments (e.g., Aczel et al., 2020; APA Style JARS) lay solid foundations for clearer and more transparent broad reporting practices, the planning and reporting of methodological aspects of cognitive and neuropsychological research protocols remain insufficiently addressed, and operational details crucial for the replicability of findings lack proper coverage. These studies require bespoke reporting standards due to their reliance on fine-tuned task designs (e.g., number of trials, task conditions), nuanced measurements (e.g., reaction times, error rates), technological setups (e.g., software, hardware), (neuro)psychological tests (e.g., cut-offs), and questionnaires that need to be reported with precision to ensure reproducibility and comparability across studies. Without such detailed reporting, it becomes challenging to assess the validity of findings or replicate experimental protocols. Developing tailored guidelines that address these specific needs could bridge the existing gap, fostering greater reliability and transparency in cognitive and neuropsychological research.

Here we introduce the Preferred Evaluation of Cognitive And Neuropsychological Studies (PECANS), a comprehensive checklist designed to ensure that all relevant information is included in research reports within the broad fields of cognitive psychology and neuropsychology. Its purpose is to support robust scientific findings, reproducibility of results, and enhance replication accuracy by third-party scientists. The PECANS checklist assesses all sections of the IMRAD format, enabling authors to cover in great detail, and keep track of, the wide range of critical choices they have to make while preparing a research protocol and reporting results. However, to minimize overlap with valuable existing instruments and guidelines, the PECANS checklist places relatively less emphasis on the Introduction and Discussion sections while focusing more extensively on three widely used methodologies in cognitive psychology and neuropsychology research: experimental tasks, (neuro)psychological tests, and questionnaires. Additionally, the checklist maintains a balanced length by including skippable items tailored to the relevance of each specific project.

Developed by following Moher et al. (2010) guidelines and employing the Delphi method to conduct a consensus building process among international experts in the fields of cognitive psychology and neuropsychology, PECANS focuses on ensuring high-quality study planning and reporting, offering the scientific community a tool that guides the planning, design, reporting, and checking of experimental research, thereby enhancing the replicability, rigor and completeness of protocols and results. By generating a report through its dedicated application, PECANS allows researchers to indicate whether each recommendation has been addressed in their work, facilitating a quick assessment of the paper’s quality in terms of research procedures and completeness. Additionally, PECANS will serve as a guideline to highlight best practices for research to consider when drafting and conducting a research protocol.

## Methods

### The Delphi method

The Delphi method is a structured group process that involves a series of rounds to survey expert opinion and reach a group response (Dalkey & Helmer, 1963). This approach is used to generate reliable, accurate, and insightful knowledge in situations where there is insufficient information (Hasson et al., 2000). The method involves structured group communication among a panel of international experts using a series of recursive questionnaires (Adler & Ziglio, 1996; Moher et al., 2010).

A consensus-building process among international experts was carried out using the Delphi method to discuss the best practices in cognitive, behavioral, and neuropsychological research. The primary objective of this process was to develop a consensus-based tool and guidelines that can assist researchers in all stages of experimental research and manuscript drafting. In addition, the checklist was tested through a proof-of-concept phase, as described below. The ultimate aim was to enhance research quality and improve the replicability of results.

**Fig. 1:**
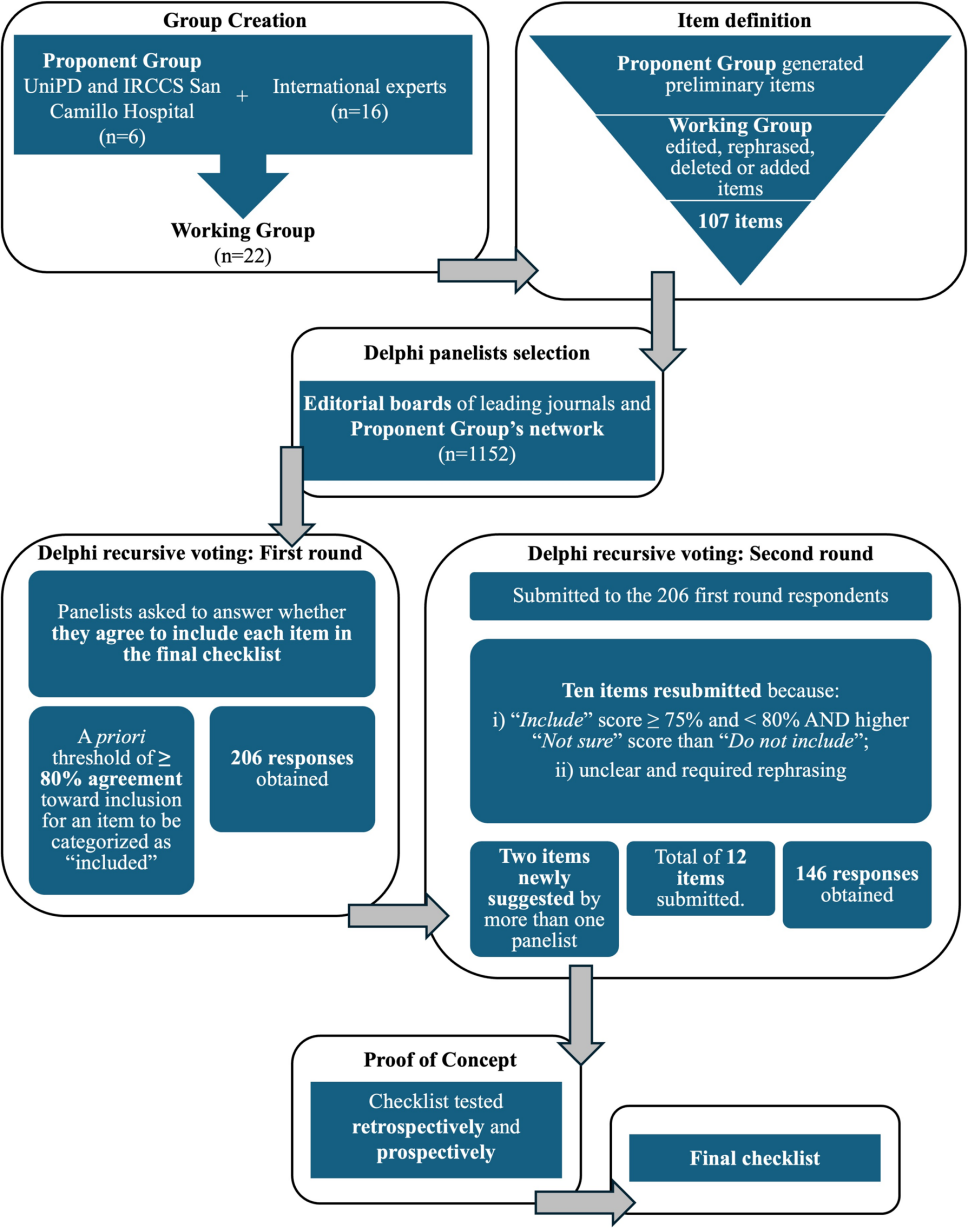
Flowchart illustrating the development process of the PECANS checklist

The Delphi process took place between 2021 and 2022, while the proof of concept through a “field usability” study took place in late 2024 and early 2025. The procedure will be described in the following paragraphs and is schematically represented in Fig. 1. All described materials have been anonymized and deposited in the associated OSF repository: 10.17605/OSF.IO/JVZE5

### Working group creation

A group of six authors from the University of Padua and the IRCCS San Camillo Hospital in Venice formed the Proponent Group. The Proponent Group reached out to international experts in cognitive psychology and neuropsychology with established expertise in the relevant fields and who have an active interest in open science practices, inviting them to participate in the project. Sixteen experts agreed to participate, forming, together with the Proponent Group, the PECANS Working Group, resulting in a total of 22 members.

### Item definition

The Delphi method typically commences with a brainstorming session, which serves to gather specific information about the topic under examination (Hsu & Sandford, 2007). Once the topic and its crucial aspects are framed, the technique involves creating a well-structured questionnaire or set of items to be submitted to panelists for voting.

To achieve this, the Proponent Group from the University of Padua and the IRCCS San Camillo Hospital in Venice generated several items during a brainhack initiative (Gau et al., 2021). These items were designed to gather “*Yes*”, “*No*”, or “*Not applicable*” responses from the checklist’s final users. The preliminary items were then shared with the PECANS Working Group to solicit feedback and suggestions. A document was circulated among the Working Group with a request to edit the items, rephrase them if necessary, delete or add items, and provide feedback. Following this process, a final list of 107 items covering all aspects of a research protocol and manuscript drafting was created and unanimously approved by all authors. Items were divided into sections and subsections, as follows: *Introduction* (five items); *Methods – reproducibility* [Pre-registration (three items); Study design (nine items); Participants (12 items); Ethical issues (eight items); Apparatus, Instrumentation, and Setting (four items)]; *Methods – experimental task* (11 items); *Methods – (neuro)psychological tests* (12 items); *Methods – questionnaires* (ten items); *Methods - Other* (two items); *Statistical analyses* (eight items); *Results* (seven items); *Discussion* (four items); *Additional information* (two items); *Data availability* (11 items).

### Selection of Delphi panelists

As explained above, the Delphi method requires the generated questionnaire or set of items to be submitted to panelists for voting. The selection of panelists can affect the quality of the results generated, and there is no clear definition of a “Delphi panelist” (Hsu & Sandford, 2007; Welty, 1972). To overcome this issue, panelists were chosen based on the relevance of their background and expertise to the project’s objectives (Belton et al., 2019; Hsu & Sandford, 2007). Particularly, the Proponent Group identified 1152 potential panelists by selecting all members of the editorial boards of leading scientific journals in behavioral and cognitive psychology and neuropsychology, as well as researchers from their personal networks, all of whom held at least a PhD.

### Delphi recursive voting: First round

The items were presented in a Google form, and panelists were asked to answer whether they agree to include an item in the final checklist or not, using a multiple-choice answer format (“*Include*”, “*Do not include*”). In addition, the option “*Not sure*” was also provided to enable panelists to abstain from a binary decision for items that lacked clarity and proper construction. After the survey, panelists were allowed to provide additional comments and clarify their responses.

After the first round of voting, the Working Group analyzed the percentages of agreement and reviewed feedback. An a priori threshold of ≥ 80% agreement toward inclusion, typically applied in Delphi studies (Moiola et al., 2021; Taylor 2020; Riva et al., 2021), was set for an item to be categorized as “included” (Belton et al., 2019; Hasson et al., 2000; Keeney et al., 2001).

Based on suggestions, feedback, and potential issues raised by panelists, it was decided to conduct a second round of Delphi voting to further refine the checklist.

### Delphi recursive voting: Second round

To work on items that did not achieve a clear consensus or were deemed unclear and required rephrasing, a second round of voting was conducted. New items were also introduced, suggested by panelists following the first round.

Each panelist who participated in the first round was given a questionnaire containing 12 items, with the option to answer “*Include*” or “*Do not include*”. The option “*Not sure*” was omitted to gather a definitive agreement regarding inclusion or exclusion.

Among these 12 items, eight were already presented in the first round. These items did not reach consensus but met one of the following criteria: (i) received an “*Include*” score ≥ 75% and < 80% AND a higher “*Not sure*” score than “*Do not include*”; (ii) were considered unclear by the panelists and required rephrasing. One of these eight items was split into three as suggested in the first round, resulting in a total of ten items. The remaining two items were newly suggested by more than one panelist.

The original items identified for rephrasing underwent modifications based on panelists’ requests and were presented alongside their original form. A brief explanation of the rationale for (re)submission and (where applicable) the percentage of agreement from the first round of voting was provided alongside each item.

Once again, panelists were given the possibility to provide further comments or clarifications of their responses. The level of agreement was set again at ≥ 80% of consensus.

### Proof of concept

The checklist obtained after the second round of the Delphi recursive voting was tested on both published papers and on manuscripts in preparation to evaluate its usability, clarity, and potential redundancies. Particularly, all members of the Working Group were asked to distribute the checklist within their laboratories, applying it to both a published paper and a manuscript in preparation. The distributed checklist was structured according to its intended use, allowing users to select “Yes”, “No”, or “Not applicable” for each item. Additionally, users could indicate whether an item was ambiguous or would benefit from explanatory text and provide comments to highlight specific concerns.

## Results

### Delphi panelists

A total of 206 experts took part in the first round of voting. This included the 22 members of the Working Group and 184 panelists. Out of these, 146 experts (22 members of the Working Group and 123 panelists) also participated in the second round of voting. The panelists had the option to either sign themselves or remain anonymous. The names of those who chose to disclose their identity and completed both rounds of voting can be found in the Acknowledgements section (the PECANS Extended Working Group), as communicated to them in the invitation e-mail.

### Items

Two rounds of voting were conducted to finalize a checklist consisting of 51 items and 33 sub-items. The detailed breakdown of the voting percentage for each item in both rounds can be found in the associated OSF repository.

### Proof of concept

A total of 35 responses were collected from the testing phase, including 17 evaluations of published papers and 18 of manuscripts in preparation. Feedback indicated the need to separate items that contained multiple questions, allowing users to respond more precisely to each component. Additionally, some items required further clarification, primarily through the inclusion of examples. One item (*Did you state the type of study? Whether the study is correlational (including epidemiological or quasi-experimental) or a true experiment?* *Longitudinal or cross-sectional?*) was removed, as it was deemed superfluous.

Use of the checklist on published papers revealed that information related to only 17 items was consistently reported. Specifically, an exploratory threshold of ≥ 80% “*Yes*” responses for a given item was used to define consistent reporting. These items (1, 2, 3, 13, 18, 19, 20, 21, 29, 30, 36, 38, 39, 43, 45, 46, and 48; Table 1) pertain to rationale and hypothesis formulation, ethical considerations, demographic information reporting, analysis, and the discussion of results and limitations.

**Table 1:** The final checklist. Subitems are preceded by either “If yes” or “If not”, indicating the response they depend on. These labels are not included in the app implementation of the checklist but are provided here for clarity purposes

ITEMS
**INTRODUCTION**
1. Did you report the rationale of the study in light of available literature?
2. Did you report the aims of the study?
3. Did you report whether your study is exploratory, confirmatory, direct replication?
4. In the case of confirmatory studies, did you clarify the hypotheses or the expected results of the study by specifying the direction (e.g., positive/higher or negative/lower) of the expected relationships or differences?
4.1 If yes, did you make clear what outcomes would disconfirm the hypothesis?
**METHODS**
***Pre-registration***
5. Did you pre-register the study?
5.1 If yes, did you report whether the study has been pre-registered before data collection and analyses?
5.2 If yes, did you provide the link to the pre-registration or pre-registration number?
5.3 If yes, did you describe deviations from pre-registration?
***Study design***
6. Did you report whether the study is a between-participants, within-participants, or a mixed study?
7. If a within-participants (or quasi-experimental) design was used, did you report, if appropriate, the control conditions and counterbalancing?
8. If a control group was present, did you report it?
8.1 If yes, did you specify whether participants have been randomly assigned to the experimental and control group?
8.2 If yes, did you specify how exactly the control group is comparable/different from the treatment group?
8.3 If yes, did you specify whether the control group is active or passive? (for instance, in a cognitive training experiment, is the control group doing another training, or doing anything?)
9. Where applicable, did you report if the experiment is single blind or double blind?
***Participants***
10. Did you perform an a priori power analysis to determine sample size?
10.1 If yes, did you report all details (including the a priori effect size, the power level, the type and total number of statistical tests performed, and any other relevant assumption)?
10.2 If not, did you justify your sample size in another way?
11. Did you report the population(s) from which you sampled (e.g., nationality, whether sampled from the general population or, for instance, from a specific university track or a particular hospital)?
12. If the data are not novel, did you specify whether they have been included in previously published articles and/or deposited in online repositories?
13. Did you report demographic information or other information for each group that may be relevant for your research? Example: gender (m, f, other), age (years, min/max, mean, *SD*), education, handedness, ethnicity, etc.?
14. Did you report how the participants of the study have been recruited?
15. Did you report the criteria of inclusion/exclusion?
15.1 If yes, did you report whether inclusion/exclusion criteria were established prior to data analysis?
16. If participants received any form of compensation, did you specify the type (e.g., money, exam credits, no reward)?
17. In case of clinical research, did you report the diagnostic criteria selected (e.g., DSM-5), the instruments used to corroborate diagnosis (e.g., SCID-PD) and specific characteristics of the participants included (e.g., pharmacotherapy, disease duration)?
18. Did you report how many participants have been tested, the number and reasons for any exclusions, and the final numbers included in each statistical analysis?
***Ethical issues***
19. Did you report if individual informed consent was obtained?
19.1 If yes, if deception was used in the study, did you explicitly report this?
20. Did you report if the study has been approved by an ethical committee?
20.1 If not, did you specify why ethical approval was not requested or not obtained?
***Apparatus, Instrumentation, and Study Setting***
21. Did you report if the data collection has been done online or in person?
22. Did you report the software and hardware (where relevant for the study) used for task presentation and response acquisition?
23. If you performed an experimental task and a neuropsychological test battery or questionnaires, did you report whether all evaluations were performed on the same day?
24. Did you describe those characteristics of the experimental setting that, if manipulated, might modulate the size of the effect(s) under investigation (e.g., type of screen, room illumination, distance from the monitor, if the experimenter stays in the room, if the experimenter is a peer or an authoritative person)?
**METHODS - EXPERIMENTAL TASK**
25. Did you administer any experimental task? If yes:
25.1 Did you describe the task to perform (i.e., what the participants are asked to do in sufficient detail so that others could replicate the task)?
25.2 Did you describe those characteristics of the stimuli that, if manipulated, might modulate the size of the effect(s) under investigation (e.g., size, colour, eccentricity of the visual angle, sound intensity in dB etc.)?
25.3 Did you make explicit all experimental and control conditions and counterbalancing (if applicable)?
25.4 In block designs, did you report the number of blocks?
25.5 Did you report the number and length of breaks, if any?
25.6 Did you report the total duration of the experiment?
25.7 Did you report the number of practice trials (if any) and experimental trials?
25.8 If applicable, did you report the number of trials per block?
25.9 Did you describe the trial timeline (e.g., inter-stimulus interval; presentation of a black screen, stimulus duration) and if trial order was random, pseudo-random, or fixed?
25.10 Did you report how the instructions before doing the task were given (i.e., written or orally; in a standardized way or not)?
25.11 Did you report all variables collected (e.g., RT, accuracy, errors)?
25.12 Did you report the response effector (e.g., verbal, feet, hand, left/right side, which fingers)?
25.13 Did you report if there is response feedback or any response-contingent reward?
**METHODS - (NEURO)PSYCHOLOGICAL TESTS**
26. Did you administer any (neuro)psychological tests? If yes:
26.1 Did you report the details of the neuropsychological evaluation (e.g., cut-off scores, reference papers, norm data)?
26.2 Did you describe the test (i.e., what the participants were asked to do)?
26.3 Did you report how the test was administered (e.g., on a lab computer, tablet, online)?
26.4 Did you report the variables collected (e.g., RT, accuracy, type of errors)?
26.5 Did you report the response effector (e.g., verbal, feet, hand, left/right side, which fingers)?
26.6 Did you report the response modality (e.g., response keys, keyboard, mouse, touchscreen, clicking, swiping, joystick, dynamometer)?
26.7 Did you report whether the tests were done on the same day?
**METHODS - QUESTIONNAIRES**
27. Did you administer any questionnaires? If yes:
27.1 Did you describe the questionnaires (i.e., what the participants are asked to do)?
27.2 Did you report how the questionnaire was administered (e.g., on a lab computer, tablet, online)?
27.3 Did you report who answered the questionnaires (participant, parent, caregiver, other)?
27.4 Did you report how the questionnaires were scored or provide a reference to the questionnaire manual?
**STATISTICAL ANALYSIS**
28. Did you describe the statistical method used for all analyses and the nature of inference (e.g., null hypothesis testing, interval estimation, Bayesian analysis, predictive modelling)?
29. Did you report the relevant information for each analysis (e.g., the structure of the models, the methods used for hypothesis testing, the nature of priors for Bayesian analysis, and the nature of any feature selection and cross-validation operations used for machine learning analyses)?
30. Did you state all dependent and independent variables (including covariates)?
31. For online studies, did you report all data cleaning procedures (e.g., removal or duplicate or automated bot responses?
32. If needed, did you report whether outlier analysis was performed of what type (e.g., multivariate outliers, influential cases), and at what level of the data?
33. Did you report if any data point (e.g., practice trials, errors outliers, too-fast or too-slow responses) were excluded from the analysis and why, and to what percentage the excluded data points amounted to?
34. Did you report how missing data (e.g., dropouts) were handled?
35. Did you report how many statistical tests were performed (including subgroups analyses)?
35.1 If yes, if more than one test was performed, did you specify whether indices of evidence (e.g., p-value, CIs) were adjusted for multiple comparisons and how (e.g., Bonferroni, False Discovery Rate correction)?
**RESULTS**
36. Did you report descriptive analyses of dependent variables, including demographics: mean and standard deviation (or median and range, etc.)?
37. Did you report appropriate summary statistics for all tested effects? (including confidence intervals or other uncertainty indices)?
37.1 If yes, did you report relevant measures of uncertainty?
38. Is it clear in your analysis how your main hypotheses were tested? Can readers easily extract summary statistics for each tested hypothesis (e.g., to include in a meta-analysis)?
39. Did you report all the exact (i.e., not just p <.05) main statistics (e.g., F-values for ANOVA, degrees of freedom, r for correlation, Bayes factor for Bayesian analysis) for all tested effects (including main effects, interactions, post hoc analyses)?
40. Did you also report the non-significant results?
41. For images and tables: did you plot or report the effects and the uncertainty indices (e.g., CI, or standard error)?
42. Did you clearly specify in the figure captions which measures of uncertainty are represented by the error bars?
**DISCUSSION**
43. Did you summarize and explain the results, also the ones in contrast with the hypotheses, in relation to the hypotheses and the aims of the study (irrespective of potential limitations)?
44. In the case of direct replication, did you make clear whether the original results were replicated or not, and which type of replication has been performed?
45. Did you discuss the results taking into consideration the evidence and literature in agreement and disagreement with the findings?
46. Did you discuss the limitations of the work (reporting potential bias, e.g., bias of inclusion/sex; limitations on generalizability of the results)?
**ADDITIONAL INFORMATION**
47. Did you mention the contributions of all collaborators (e.g., following the CRediT taxonomy)?
48. Did you disclose any conflict of interest (including relevant grants)?
**DATA AVAILABILITY**
49. Did you report whether the experimental material (e.g., task, participant instructions; stimuli; video of the experimental procedure) are freely available, available upon request, or not available?
49.1 If yes, did you provide the link to the material?
50. Did you report whether the data are freely available, available upon request, or not available?
50.1 If yes, if available, did you describe the variables included in the dataset and instructions on how the data are structured?
50.2 If yes, did you provide the link to the data?
50.3 If yes, did you make clear to whom requests for data/materials are to be directed?
51. Did you report whether the code/script to perform statistical analyses is freely available, available upon request, or not available?

### The final checklist

The final checklist, presented in Table 1, consists of 51 items and 42 sub-items. Each item must be answered with “*Yes*”, “*No*”, or “*Not applicable*”. Sub-items are displayed to users based on the answer provided to the corresponding main item.

The final checklist (and a short version for Registered Reports) can be accessed and compiled through the PECANS application by following this link: https://pecans.shinyapps.io/Pecans_Checklist/.

## Discussion

In this paper, we present the PECANS checklist, a tool designed to enhance research rigor, completeness of reporting, and reproducibility. To create the PECANS checklist, we assembled a panel of international experts selected from the editorial boards of leading scientific journals specializing in the broad fields of cognitive psychology and neuropsychology. The final PECANS checklist comprises 51 primary items and an additional 42 sub-items that are conditional on the primary ones. Although the present checklist may appear relatively lengthy, it offers researchers flexibility by allowing them to skip sections that do not apply to their studies. For instance, if a study does not relate to neuropsychology or if no questionnaires were administered, researchers can omit the secondary items that are conditional upon the primary ones. In our attempt to balance comprehensiveness and brevity, we prioritized completeness, given the critical nature of the matter. Furthermore, the strong consensus among panelists, as evidenced by their acceptance of 72% of all proposed items during the first round of voting, underscores the perceived importance of incorporating a wealth of information to enable rigorous, transparent reporting and reproducible methods.

Intriguingly, certain items garnered near-unanimous agreement, such as the questions concerning the clarity of hypothesis testing and the ease of extracting effect sizes and statistics for each hypothesis (97% consensus). In contrast, certain items relevant for assessing the strength of results in contemporary research (Button & Munafò, 2017; Lakens, 2013) hardly reached the predefined threshold for acceptance. For example, the item regarding the necessity of reporting an a priori power analysis to determine sample size reached only 80% consensus. Testing of the instrument on published papers (during the proof-of-concept phase) further revealed that this information is severely underreported, as 76% of the published papers assessed did not include a power analysis. Moreover, among the manuscripts that omitted this information, more than half (53%) did not justify their sample size in any other way. Other items initially fell short in the first round, but gained acceptance in the second round of voting, as for the item asking to provide a link to study materials (79% consensus in the first round, 91% in the second one), or the one asking to state the type of study, meaning correlational (e.g., epidemiological or quasi-experiment), experimental, longitudinal or cross-sectional (78% consensus in the first round, 86% in the second one). This last item, however, was removed after the proof-of-concept phase, as it was deemed superfluous given that the information could be easily inferred from the Methods section of a manuscript. Other items that are considered highly relevant for assessing the strength and reliability of results, such as effect size/uncertainty of effects (63% of consensus) (Lakens, 2013), were excluded from the checklist as they did not meet the threshold for acceptance.

It is crucial to acknowledge that the checklist we present reflects the consensus of 146 panelists. Although this number is indeed sufficient for a successful Delphi panel (Akins et al., 2005; Belton et al., 2019; Steurer, 2011), it is important to recognize that the overall community of scientists involved in research on cognitive psychology and neuropsychology may not be fully represented. As a result, some crucial items may have been omitted, or items covering information that is becoming increasingly important may not have been sufficiently addressed, given the continuous evolution of reporting standards. Although the PECANS checklist is designed to cover all sections of the IMRAD format, providing authors, researchers, and other stakeholders with comprehensive guidance in a single instrument, we recommend that end users supplement it with existing instruments (e.g., STROBE guidelines for observational studies in epidemiology (Von Elm et al., 2007), CONSORT guidelines for randomized trials (Schulz et al. 2010), CARE guidelines for case reports (Riley et al. 2017), COBIDAS for best practices in MRI data analysis (Nichols et al., 2017), ContES guidelines for tms-fMRI studies (Ekhtiari et al., 2022)) as needed, depending on the specific requirements of the study.

The PECANS checklist is intended to serve as a practical resource for various stakeholders in cognitive psychology and neuropsychology research. Ideally, PECANS could establish a standard for reporting research outputs in these fields, similar to how PRISMA has done for the reporting of systematic reviews (Moher et al., 2009). By enhancing the replicability, rigor, and completeness of protocols and results, PECANS addresses the growing need for transparency and consistency in research practices. Researchers, in particular, can benefit from this instrument by having access to a structured framework that ensures best practices are followed throughout the study lifecycle. Through its dedicated application, PECANS enables researchers to quickly assess whether each recommendation has been addressed, facilitating a rapid evaluation of the paper’s quality in terms of research procedures and completeness. This process not only helps identify gaps in methodology but also promotes transparent reporting, which increases the likelihood that findings will be reproducible and robust. Furthermore, PECANS serves as a valuable guideline for researchers when drafting research protocols, ensuring adherence to established standards from the outset.

Peer reviewers also benefit from PECANS, as it provides a clear checklist to assess the rigor and transparency of methods and results in submitted manuscripts. The structured evaluation process ensures that reviewers apply consistent criteria, helping them provide more focused and detailed feedback. Journal editors can similarly use PECANS as a standardized tool to assess the quality of submitted manuscripts. The checklist allows editors to quickly determine whether key methodological and reporting standards have been met, thereby making the peer review process more efficient and consistent.

Institutions and agencies can use PECANS to foster an environment where research quality is consistently prioritized, thus supporting the advancement of rigorous and reproducible science, whereas for early-career scholars, PECANS would be a particularly valuable learning tool, offering clear, practical guidance on the essential components of high-quality research.

In summary, PECANS – along with its shorter version for Registered Reports – is a versatile tool that not only improves the quality and transparency of individual research projects but also fosters a broader culture of rigorous scientific inquiry across the academic and research community, a critical goal in today’s research landscape (Nosek et al., 2022).

## Conclusion

We have developed a consensus-driven checklist comprising essential components required for enhancing rigor, quality, completeness, and reproducibility of studies, and study reports, in cognitive psychology and neuropsychology research. Users can readily access the agreed-upon, tested version (and the short version for Registered Reports) on the PECANS application by following this link: https://pecans.shinyapps.io/Pecans_Checklist/.

## Data Availability

Anonymized raw and preprocessed data from both voting rounds and the proof-of-concept phase are publicly available on the project’s Open Science Framework page, as well as all survey materials: invitation and follow-up e-mails, preliminary items with anonymized co-authors’ comments, Google Forms from both voting rounds, and anonymized comments from panelists (Delphi panels) and testers (proof-of-concept phase). OSF: 10.17605/OSF.IO/JVZE5
